# Novel Biomarkers for the Assessment of the Cardio-Renal Syndrome—A Paradigm Shift

**DOI:** 10.3390/jcm12155116

**Published:** 2023-08-04

**Authors:** Yacov Shacham

**Affiliations:** Department of Cardiology, Tel-Aviv Sourasky Medical Center Affiliated to the Faculty of Medicine, Tel-Aviv University, Tel-Aviv 64239, Israel; kobyshacham@gmail.com

Among patients admitted to medical wards, a growing number have various degrees of cardiac and renal dysfunction. A diseased heart has numerous adverse effects on renal function but, at the same time, renal insufficiency can significantly impair cardiac function.

It is well known today that the direct and indirect effects of insults to either of these organs can initiate and perpetuate combined disorder in both of them, through a complex combination of neurohormonal feedback mechanisms; primary disorders of one of these two organs often result in secondary damage to the other. These bilateral interactions set the pathophysiological basis for a clinical entity known as “cardiorenal syndrome” (CRS). Although originally used to define a condition characterized by the occurrence of renal insufficiency secondary to failure, the term, CRS, is now used to describe the adverse effects of impaired renal function on the cardiovascular system. Recent data have improved our understanding of the crosstalk between these organs and have highlighted the efficacy of specific therapies in attenuating combined organ damage.

Numerous biomarkers of inflammation are known to rise in the event of, and may be highly valuable in the prediction of, both adverse cardiovascular and renal outcomes. However, only a small number of these biomarkers have specifically addressed the unique cardio–renal interaction. Neutrophil gelatinase-associated lipocalin (NGAL) is an early and sensitive marker of acute kidney injury (AKI) [1,2,3]. Recent evidence has also suggested the possible role of NGAL as an inflammatory modulator [4,5]. In this Special Issue of the *Journal of Clinical Medicine*, which focuses on new insights into cardio–renal interactions, includes several reports demonstrating the unique role of NGAL in the prediction of combined renal and cardiovascular adverse outcomes in acute coronary syndrome patients. Zhaler et al. demonstrated a cohort of myocardial infarction patients in whom serum NGAL levels were collected 24 h post-admission [6]. In-hospital adverse outcomes were increased in the high-NGAL group, who exhibited higher rates of AKI, worse cardiac systolic function, and higher 30-day mortality rates. Their results also demonstrated that NGAL was independently associated with cardiovascular outcomes and performed better than traditional inflammatory markers (e.g., C-reactive protein, leukocyte count, etc.). A second report by Højagergaard and colleagues included a cohort of >1600 patients with myocardial infarction [7]. They demonstrated that an NGAL plasma level above the median was an independent marker for severe AKI and all cause death within 30 days. Their analysis also indicated that the predictive value of NGAL plasma levels was higher within 6–24 h of admission.

Recent guidelines recommend the use of new stress damage biomarkers in clinical practice for the prevention and management of AKI episodes [8]. Biomarkers are unique tools which may be used to distinguish between various AKI phenotypes and may facilitate risk stratification, independent of an AKI event [9]. Biomarkers of renal function and damage may have an additive value for both diagnostic and prognostic information based on serum creatinine, providing additional information on the pathophysiology of the index event. Such information can be further implemented to improve AKI risk prediction and clinical decision making. The report by Banai et al., the first conducted on a population of myocardial infarction patients, incorporated contemporary recommendations for AKI diagnosis [9]. They used NGAL to detect structural renal damage, while functional AKI was determined using the KDIGO criteria, based on changes in serum creatinine. Patients were stratified into four AKI phenotypes: no AKI, subclinical (pseudo) AKI, hemodynamic AKI, and severe AKI. A unique, downstream sequela was demonstrated, based on the AKI phenotype, with a gradual increase in the risk of adverse outcomes between patients with subclinical AKI, hemodynamic AKI, and severe AKI. It thus appears that AKI is a heterogeneous condition consisting of distinct phenotypes. The utilization of biomarkers may aid in overcoming the known drawbacks of serum-creatinine-based AKI definitions, thus improving AKI phenotyping and informing the selection of therapies.

Several important limitations should be noted regarding the utilization of this specific biomarker: NGAL levels measured following PCI may be subject to contamination with contrast fluid and could lead to direct damage to the kidney tissue (i.e., ischemia-reperfusion injury following PCI). In order to evaluate the possible prognostic value of NGAL independently of AKI, NGAL levels prior to admission are needed in order to make an accurate assessment of the inflammatory state. In addition, urine NGAL levels, compared to plasma NGAL levels, may be a more precise indicator of renal damage and thus, may be more suitable for such an assessment. It is also possible that additional blood sampling, as inflammation peaks, would be of value, possibly providing a more accurate indication alongside other inflammatory biomarkers.

In conclusion, cardio–renal interactions are complex and often bidirectional in nature. Among the various biomarkers, NGAL may provide new insights into cardio–renal crosstalk. Further investigations assessing the possible role of this novel biomarker in the assessment of cardio–renal interactions are warranted.
